# Repertory of the Back

**Published:** 1898-07

**Authors:** E. H. Wilsey

**Affiliations:** Parkersburg, W. Va.


### REPERTORY OF THE BACK.

E. H. Wilsey, M. D., Parkersburg, W. Va.

(Continued from June number, page 257.)

### SPINE.

**Sensitive,** painful sensitiveness of spine from last cervical vertebra to fifth dorsal, Tellur.

— the whole spine very sensitive to touch, even by a sponge or leaning against a chair, Agari.

— sensitiveness, especially in cervical and upper dorsal region; soreness of muscles, | Cimicif., | Agar.

— spine very sensitive, pains shooting into head, | Cinch.

— very sensitive, and hyperæsthesia of spine from sixth cervical vertebra to small of back, slightest touch intolerable, | Cup.

**Sensitiveness** of spine to touch, | Zinc.

— of dorsal and cervical vertebræ, | Carduus.

— of last cervical vertebræ and first dorsal to pressure, || Chin-sul.

— spinal irritation, great sensitiveness between vertebræ; sits sideways in chair to avoid pressure against spine, | Therid.

— great, of cervical vertebræ to pressure, | Arn., Stram.

— deep-seated, sensitive pain sticking in ilium of one or the other side, an inch to an inch and one-half from spine, extending obliquely inward toward sacrum, | Berb.

— great, of cervical vertebræ to touch, | Hyperi.

— great, between vertebræ, | Nat-m.

— through vertebræ, painful shrieks when they are touched, cannot sleep on back, | Kali-c.

**Sharp,** sudden stitches beside the spine, darting forward through the chest into cartilages of the left ribs in the evening, Mez.

— sensation, as if atlas and axis had been pried apart; on rising to feet it changed to sharp pain, Curare.

— pain at lower part of sternum, passing through to spine or lower angle of right scapula, Elater.

— frequent, sharp stitches, like from splinters on dorsal vertebræ, Agar.

SPINE.

**Sharp,** pains in lower spine, Ol-jec.
— pains in whole length of spine, better lying on back, Zinc.
— pains from upper dorsal into occiput, | Kali-c.
— pains in spine, shooting into occiput (confinement), Petrol.
**Shooting** pains in single vertebræ, Agari.
— violent shooting, burning pains, deep in spine, | Agar.
— from dorsal spine half-way to the left side, Rumex.
— violently between shoulders and in whole of back, worse on inspiration, with tension in spine when moving the trunk, also in evening; relieved when walking, Nat-c.
— in spine, Aco., Bell.
— in lumbar region, from vertebræ to crest of ilium, right side, Chromic-ac.
**Sitting,** weakness and lack of mobility in the spine; it feels as if it would collapse on long sitting. Bar-c.
— along spine and from sacrum down through the legs into feet; worse while sitting, Kobalt.
— pain, as from an ulcer under the skin, on the lower end of the spine, mostly when sitting and lying, Carbo-a.
— pains in middle of spine when sitting, | Arn.
— bruised pain in spine when sitting, Sabad.
— pulsation in spine when sitting, Thuja., Curare.
— while sitting pain on either side of dorsal spine, similar to that felt in chest when it is said "the food has lodged" between scapula and lumbar region, | Kobalt.
**Sneezing,** sudden sprained pain on sneezing, then drawing pain close to spine, extending into left groin and testicles; worse rising from a seat and walking, Sul.
**Softening** of spinal cord, congestion or irritation, spinal meningitis, myelitis or spinal paralysis, Crotal.
— of spinal cord, loss of power, stumbles and trips, | Kali-phos.
— of spine, with curvature, Merc-c., || Sul.
— limbs, paralyzed from spinal softening, Stram.
— of spine, | Phos.
**Soreness,** up and down spine and neck, Nat-s.

SPINE.

**Soreness,** aching soreness in all the vertebræ; worse by motion and by pressure on spinous processes, | Chel.

— in lower cervical vertebræ, Con.

— stiff, sore feeling down right side of spine, then across loins and over right hip-joint, Sil.

— in a spot on right side, near spine, below loins, from rising in morning till bed-time, with sensitiveness to touch, Lith-carb.

— and pain down cervical vertebræ, | Ham.

— of spine; does not wish it touched, Iodoform.

— spine sore and painful, | Merc-jod-rub.

— with weakness of spine, Diosc.

— and pain the whole extent of spine from below upward, Eup-pur.

— in cervical spine on left side, || Sul.

— in last dorsal and first lumbar vertebræ, Bell.

— in lower part of spine, almost traceable to sciatic nerve, Brach.

**Squeezing,** pressive pain beside the lowest part of spine, Carbo-v.

**Stabbing,** in vertebræ, as with a knife, from without inward, Bell.

— from third cervical vertebræ to fifth dorsal, striking forward through chest to sternum, worse from motion, | Kali-bi.

**Standing,** pressure along spine while walking in open air; disappearing while sitting or standing, Mur-ac.

— stitches in middle of spine, on walking in open air; better when standing, jerking in sacrum, and at same time above ankle, Calc-ac.

— tired pain in lower part of spine, causes nausea and faint feeling when standing, Sep.

**Sticking,** in middle of spine, Plumb.

— through spine to pit of stomach, Ratanhia.

— in right side of spine in morning on breathing deeply, Nat-phos.

— in spine and left side of nape, Sul-ac.

— in middle of spine, better by motion, Cina.

— gnawing, with sticking in middle of spine, Helleb.

— boring, sticking in spine just below shoulders, Helleb.

— pain in spine, worse by bending back, Jambos.

SPINE.

**Sticking,** burning in spinal cord, then sticking and boring between scapulæ, better on motion, Mag-m.

— in spine, between scapulæ, Merc.

— sudden sticking near spine, extending through chest into left costal cartilage anteriorly in the evening, Mez.

—intermittent sticking along left side of spine, and in middle of trunk, Moschus.

— in middle of spine, on breathing, Dulc.

— in the whole of spine, Elaps.

— along spine mornings on waking, better moving about, Euphrasia.

— with every expiration in lumbar vertebræ, Sul.

**Stiff,** sore feeling down right side of spine, then across loins, and over right hip-joint, Sil.

— cervical portion of spine stiff, any motion thereof causing disagreeable tension and dull pain deep in, which extends towards both shoulders, | Dulc.

— neck, pain all down spine, | Cedron.

**Stiffness** of the spine, Nit-ac., Æsc-h.

— of spine and lower limbs, Tabac.

— of spine, extending up from the coccyx., Ars.

— painful stiffness of spine, with lazinesss and heaviness of the legs in the morning on walking and after rising, Calc-c.

— in spine, as after standing bent for a long time, Thuja.

— and pain down spine, with inclination to bend forward; as hard to sit up straight, Physos., Calab.

— from lower part of spine, aching pain upward with stiffness, worse in damp weather, | Nux-m.

— of back; spine becomes bent, | Carbo-v.

— after painful stiffness of spine, determination of blood to head, with ebullitions, | Calc-c.

— painful stiffness of spine and back, making change of posture very difficult, | Calc-c.

— painful stiffness of spine in morning on rising, with indolence and heaviness of legs, Calc-c.

SPINE.

**Stiffness** of dorsal spine, want of mobility, Bar-c.
— of spine, rigid in cerebro-spinal meningitis, **I** Cic.
— of spine, rigid, motion exceedingly painful, Lob-c.
— of spine, with periodic attacks of pain through chest, **I** Cepa.
— of spine, with inability to straighten after stooping, **I** Kali-b.
— of vertebræ, Coccul.
**Stinging** in cervical vertebræ, Lyss.
**Stitches,** tearing from spine to anterior spinous process of left ilium when sitting, Drosera.
— in spinal column, during motion, Meph.
— in upper part of back, along spine from below upward, or in whole back and nape of neck, with stinging sensation in right arm, as if gone to sleep, Lach.
— in spine and shoulders, China.
— in spine., Berb., **I** China., Nitr-sp-d., **I** Ran-b.
— sudden and tearing pains on each side of spine, in back and lumbar region, Cinnab.
— frequent sharp stitches, like from splinters, on dorsal vertebræ, Agar.
— in spine while sitting, is suddenly overcome by anxiety, Ruta.
— severe stitches from thoracic cavity through the spine out between scapulæ, Calc-c.
— broad stitches, as from a knife, under scapula on left side, near spine, Cup.
— painful stitches in middle of spine when respiring, Dulc.
— sharp, sudden stitches beside the spine, darting forward through the chest into cartilages of the left ribs in the evening, Mez.
— uninterrupted stitches in the spine all the day at various hours, Phos.
— small, violent jerking stitches in middle of spine, Phos-ac.
— frequent stitch-like electric shocks in a zig-zag course along spine from lumbar to scapular region, Euonymus.
— pressing stitches along spine, mostly in sacrum, with dyspnœa, Tarax.

SPINE.

**Stitches,** cutting stitches at ends of right ribs, near spine ; worse on curving back, Arg-m.

— from spine through upper abdomen to pit of stomach, Thuja.

**Stooping,** violent pain in spine, as from sudden rising after long stooping, | Arn.

— pain near spine as if a plug were forced inward two inches below left scapula on stooping, Prunus.

— pain along the spinal column ; worse on stooping, Agar.

— when stooping, spine pains as if too weak to support back, | Agar.

— drawing in spine extending upward on stooping, | Sul., Sep.

— downward drawing and pain in spinal cord, worse by stooping or bending, Daphne.

**Strain,** spine pains in the renal region or stretching as from a strain in lifting, Calc-c.

**Stumbles,** softening of spinal cord, loss of power, he stumbles and trips, | Kali-phos.

**Swelling** of left side of cervical vertebræ, Sul.

**Tabes Dorsalis** (atrophy), from sexual excesses, | Kali-brom.

— — Secale, | Sul.

— — progressive locomotor ataxia, Æsc-h., | Alum., | Arg-nit., | Atrop., | Bell., | Calab., | Caust., | Coccul., Colch., Crotal., Fluor-ac., | Gels., | Kali-br., | Lach., | Nit-ac., | Nux-m., || Nux-v., || Phos., | Phos-ac., | Pic-ac., || Plumb., | Psor., | Rhus., Sec., | Sul., | Stram., | Sil., Tarant., | Zinc.

— — from exhaustion, || Pic-ac.

— — with paralysis, | Sil.

— — from sexual excesses, | Kali-br.

— — with great excitement of sexual system, | Fluor-ac.

— — in women with weakness in legs and back, | Graph.

— — from excessive loss of fluids and semen, | Phos.

— — early stages of locomotor ataxia, especially when occurring from exposure to cold or from sexual excess, | Nux-v., | Calc-c.

— — incipient locomotor ataxia, | Caust.

SPINE.

**Tabes Dorsalis** with paralysis, | Sil.
— — with dulness of sense, | Graph.
— — cerebro-spinal exhaustion from overwork, || Phos-ac.
— — with chest pain, torpor or numbness of genitals, | Graph.
**Tearing** in spine, Berb., | Caps., | Cina.
— jerking pain in middle of spine, | Cina.
— and drawing down spine, | Cina.
— sudden stitches and tearing pains on each side of spine in back and lumbar region, Cinnab.
— between scapula in evening before lying down and in spine, | Coccul.
— to left of spine: and behind right axilla, Colch.
— below scapulæ near spine, Lyc.
— in lower part of spine, causing shuddering so that she bent backward, then stitches at 5 P. M., Mag-carb.
— in whole spinal column from above downward, Mang.
— stitching from spine to anterior spinous process of left ilium, when sitting, Drosera.
— and drawing down right side of spine from axilla to the last rib, Guaj.
— burning, tearing on right side of the spine above sacrum, | Kali-c.
— downward on the whole spine, in rest and in motion, Mang.
— deep in spine toward left side, Ign.
— in spine when stooping or bending backward, also when walking, | Chel.
**Tenderness** of spinal column when stretching, Med.
— great on pressure over posterior, spinous processes of all cervical and first four dorsal vertebræ, | Coloc.
— of spine worse in cervical and lumbar region, Tabac.
— of dorsal region of spine to pressure, Plumb.
— of spine on pressure, Plant., | Sep.
— in whole spine, most in neck below occiput, | Lach.
**Tension** and drawing in spine, | Nat-m.
— in dorsal vertebræ, Aur-mur.

SPINE.

**Tension** in, painful followed by loss of consciousness, **|** Nat-m.

**Tensive** rheumatic pain in spine, Zinc.

**Throbbing,** intermittent in left side, near spine, Dulc.

— intermittent on right side of spine, near 11th and 12th dorsal vertebræ at 11 P. M., worse by motion, extending by single jerks into right thigh, Canth.

— drawing, burning, and throbbing pain in spine, Bell.

**Touch,** cervical vertebræ very sensitive to touch, **|** Hypericum.

— shrinks from least touch on spine, between cervical and lumbar region, **|** Hyper.

— sore spine, does not wish it touched, Iodoform.

— sensitiveness of vertebræ to touch, but cannot locate pain, **|** Coccul.

— a slight touch along spine provokes spasmodic pains in chest and indescribable distress in cardiac region, at times heart feels as if twisted, Tarentula.

— the whole spine very sensitive to touch, even by a sponge or leaning against a chair, Agar.

— a large portion of spine so sensitive to touch that she cried out, turned pale, and was seized with nausea, belching, and retching, **|** Atrop-s.

— sensitiveness of spine to touch, **|** Zinc.

— pressive pain in spine, between scapulæ, with short breath, worse on respiring, with pain in spinal vertebræ on being touched, Calc-c.

— could not bear least touch along whole vertebræ, Lyss.

— slightest touch on vertebræ produced an irritability akin to convulsions, Lyss.

— three upper dorsal vertebræ, very painful and sensitive to touch, **|** Chin-s.

**Traumatic** meningitis, **|** Hyper.

**Twinges** near dorsal vertebræ, **|** Amyl.

— of spine, with numbness of feet and hands, **|** Calab.

— numbness of feet and hands and other parts of body, burning and twingeing sensations referable to the spinal column, **|** Physos.

SPINE.

**Twitching** in spine, with great uneasiness, | Agar.
— myelitis with paraphlegia of extremities, of bladder and arms, with tendency to twitching and shocks, | Merc.
— over left rib near spine, then drawing pain between scapulæ, Lob-i.
— pain down spine, with twitching, Morphinum.
**Uneasiness** great in spine, | Agari.
— in lumbar vertebræ, | Sabina.
— in spine, with headache, | Agar.
**Up**, pricking up and down spine, Jug-cin.
— sensation as if quicksilver moved up and down spine, | Phos.
— cramp-like stitches up and down spine, Physos.
— numb, tired pains up and down spine and in head, | Curare.
— a sense of warm air streaming up spine into head, | Ars., Carbo-v.
**Urinate**, spinal anemia, ineffectual attempt to defecate or urinate, | Nux-v.
**Vaccination**, spinal curvature after vaccination, Thuja.
**Walking**, pain in spine when walking, then drawing pressure as if bruised, better on pressure, Ver-a.
— periodically returning insupportable pains in spine, which prevent walking, | Phos.
— pressure along spine while walking in open air, disappearing while sitting or standing, Mur-ac.
— violent shooting between shoulders and in the whole of back, worse on inspiring, with tension in spine, when moving the trunk, also in evening, better when walking, Nat-c.
— stitches in middle of spine on walking in open air, better when standing, jerking in sacrum and at same time above ankle, Calc-ac.
— aching pain in small of back or spine, worse when sitting, better by rising, walking, or lying down, Cobalt.
— slight drawing pain along spine in afternoon, changes to a seated, dull tearing in joints of legs, worse by walking, Stront.
— tired in spine when walking, so that lower part of spine feels broken, Sep.

SPINE.

**Walking,** shooting from dorsal spine, midway to left side, on beginning to walk in the room, then in a spot immediately below it in the loin, Rumex.

**Warmth,** rising from along the spine, | Lyco.

— extending to head from spine, Can-ind.

— in spine up to the neck, Carbo-v.

**Weakness** in dorsal region of spine, with intermittent prickings, Raphanus.

— aching along spine, especially between scapulæ and in lumbar region, with weakness across loins, Arum-drac.

— of spine, with soreness, Diosc.

— great weakness in the whole spine, Hepar.

— of spine in evening, Alum.

— peculiar and great weakness on both sides of spine, she could not lie on back, Apis.

— drawing along spine and calves, with weakness in feet, Thuja.

— spine weak and nervous, | Nat-m.

— spinal weakness and debility, with difficult evacuation of scybalous stools, | Nux-v.

— pain along spine in lumbar region, with weakness and straining, Iodoform.

— spine affected after pollutions, excessive weakness shows itself in legs, | Sabadilla.

— in spine, | Æsc-h., Agari., | Cast-eq., Hep., | Phos.

— in bending, the spine aches as if it were too weak to support back, Agar.

— and lack of mobility in the spine, it feels as if it would collapse on long sitting, Bar-c.

— of spine, from exhaustion, | Gels.

— of spine, giving rise to great fatigue, exertion, and frequent desire to urinate, especially in the morning, | Phos-ac.

— after typhoid fever, fears he will be paralyzed, Selen.

— of spine, with partial paralysis, | Nat-m.

**Wince,** pressure of finger between vertebræ causes patient to wince, | Physos.

